# Arsenic Exposure, Dermatological Lesions, Hypertension, and Chromosomal Abnormalities among People in a Rural Community of Northwest Iran

**DOI:** 10.3329/jhpn.v28i1.4519

**Published:** 2010-02

**Authors:** Saeed Dastgiri, Mohammad Mosaferi, Mohammad A.H. Fizi, Nahid Olfati, Shahin Zolali, Nasser Pouladi, Parvin Azarfam

**Affiliations:** ^1^ School of Medicine and National Public Health Management Centre; ^2^ School of Public Health and Nutrition; ^3^ Tabriz University, Tabriz, Iran; ^4^ Academic Center for Education, Culture and Research, Tabriz, Iran; ^5^ Azarbaijan University of Tarbiat, Moallem, Tabriz, Iran

**Keywords:** Arsenic, Cross-sectional studies, Dermatological lesions, Hypertension, Chromosomal abnormalities, Water pollution, Water supply, Iran

## Abstract

Chronic exposure to arsenic compounds is one of the major public-health problems in many developing and some developed countries. The aim of this study was to investigate the effects of chronic exposure to arsenic on dermatological lesions, hypertension, and chromosomal abnormalities among people in a community in the northwest of Iran. The occurrence of dermatological lesions, hypertension, and chromosomal abnormalities was investigated in two groups: Ghopuz village, including 101 subjects with chronic exposure to arsenic in drinking-water and Mayan village, including 107 subjects with no exposure. Daily/yearly absorbed amounts of arsenic were calculated for all subjects. Cumulative arsenic index for each individual was then estimated on the basis of age, water consumption, and location of residence. Arsenic concentration in drinking-water sources in Ghopuz and Mayan villages was 1031±1103 μg/L and non-detectable respectively. The mean systolic blood pressure in the exposure group [n=137, 95% confidence interval (CI 132–142)] was significantly higher than that in the control group (n=107, 95% CI 99.9–114). A similar significant difference was observed for diastolic blood pressure (exposed: n=82, 95% CI 79–85 vs non-exposed: n=71, 95% CI 66–75). The incidence of hyperkeratosis was 34 times higher among the exposure group compared to the control subjects [odds ratio (OR)=34, p<0.001)]. A significant difference was also observed in the occurrence of skin-pigmentation between the two groups (OR=2.4, p<0.007). Location and severity of the pigmentations were statistically different between the two groups. Twenty-five percent of the subjects in the exposure group showed chromosomal abnormalities (p=0.05). Arsenic exposure was a serious health problem in the region. More studies are needed to investigate the long-term effects and dose-response relationship of arsenic in the region and similar areas. Wide-ranging monitoring programmes for drinking-water sources should be implemented by public-health authorities.

## INTRODUCTION

Arsenic is a hazardous, naturally-occurring element widely distributed in the crust of the earth. Inorganic forms of arsenic (pentavalent and trivalent forms) can be found in small amounts in the atmosphere, groundwater, and surface-water. Exposure to arsenic compounds is a major concern to public health in both developing and developed countries (1, 2). Arsenic is an ingredient of a wide variety of products in manufacturing industries, i.e. wood preservatives, herbicides, insecticides, pesticides, fungicides, high-emitting diodes, semi-conductors, etc., thus making workplaces a source of inhalation of, and dermal exposure to, arsenic. Arsenical drugs have also been used for treating some medical conditions (3, 4). However, the main source of high exposure of general population to arsenic compounds is water. Arsenic in drinking-water above the accepted standards demonstrated in many countries is a global problem affecting countries on all five continents (5). In some countries of Asia, the issue of chronic arsenic intoxication seems to be a more important public-health problem than in other regions of the world (6). In Asia, arsenic has been reported in groundwater in Bangladesh, Cambodia, China (including provinces of Taiwan and Inner Mongolia), India, Iran, Japan, Myanmar, Nepal, Pakistan, Thailand, and Viet Nam (5). The most serious damage to health has taken place in Bangladesh and West Bengal, India (5, 7).

In Iran, naturally-occurring arsenic is responsible for contamination in drinking-water. Kurdistan, a western province of the country, is having a major problem of arsenic contamination (8, 9).

A recent study in the villages of Hashtrud county, in the northwest of the country, showed that arsenic exists in the drinking-water at a higher level (10) than the World Health Organization (WHO) guideline value of 10 μg/L (11) and the national Iranian standard value of 50 μg/L (12) in a quarter of the villages in this area.

The acute toxicity of arsenic at high concentrations has been known about for centuries. A strong adverse effect of low arsenic concentrations was recently discovered to be associated with long-term exposure. Drinking-water is now recognized as the major source of human intake of arsenic in its most toxic (inorganic) forms (5).

A number of studies have been reported to assess the effects of arsenic-contaminated food (13) and water in those communities with high level of contamination (13–18).

The disease symptoms caused by chronic arsenic ingestion are called arsenicosis and develop when arsenic-contaminated water is consumed for several years (5).

Although some adverse effects on health have been reported for chronic arsenic exposure, it varies by different population groups, age, gender, cumulative dose of arsenic, nutritional status, genetic factors, lifestyle, individual susceptibility, and different chemical forms of arsenic in drinking-water (19–22).

Many studies have been reported on the relationship of chronic arsenic exposure with cardiovascular diseases (23–24), cerebrovascular events (25, 26), hypertension and peripheral vascular disorders (27–32), Blackfoot disease (33), carcinogenic effects (34–43), diabetes (44, 45), some neurological diseases (46–47), skin disorders (48–50), and chromosomal abnormalities (51–56).

The first visible symptoms caused by exposure to low arsenic concentrations in drinking-water are abnormal black-brown skin-pigmentation known as melanosis and hardening of palms and soles known as keratosis. If the exposure continues, skin-depigmentation is started, resulting in white spots that look like raindrops (medically described as leukomelanosis). Palms and soles further thicken and painful cracks emerge. These symptoms are described as hyperkeratosis and can lead on to skin cancer (57).

The aim of this study was to investigate the effects of chronic exposure to arsenic on dermatological lesions, hypertension, and chromosomal abnormalities in a region in the northwest of Iran.

## MATERIALS AND METHODS

### Setting and subjects

East Azerbaijan province is located in the northwest of Iran with a cool and dry climate (Fig.) that covers an area of approximately 47,830 sq km. It has a population of around four million. The province has common borders with the current Republics of Azerbaijan, Armenia, and Nakhchivan (58). This cross-sectional study was carried out in Ghopuz village (chronically exposed to arsenic) and Mayan village (control group). Both the villages are located in the East Azerbaijan province.

**Fig.**
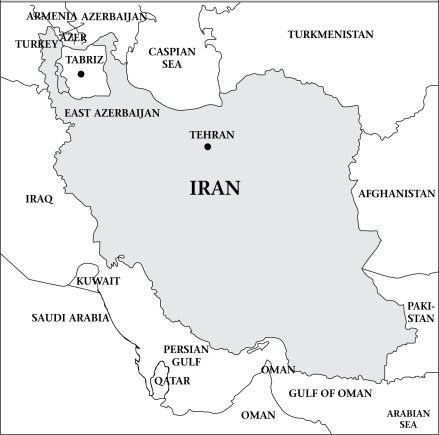
Location of study area on map of Iran

Selected villages were similar, considering the ethnic, cultural background, and other socioeconomic factors. In Ghopuz, occupation of all people was agriculture. However, in Mayan, there were other occupations along side agriculture because of less distance to Tabriz (capital city of the province).

In Ghopuz, all people aged six years and more were included as exposure group (total number of subjects was 101—48 male and 53 female). The reason for selecting subjects aged ≥6 years was the status of exposure: exposure of subjects had been removed by local water and wastewater authorities since five years before our study. In the meantime, those people aged ≥5 years were allowed to have at least one year of exposure to arsenic in their drinking-water. We, therefore, included everyone aged ≥6 years for this study. This means that every subject (aged ≥6 years) in our study had at least one year of exposure to arsenic. For further uniformity, same criteria were considered in the control village. The control group was selected from Mayan with no arsenic in drinking-water as the main selection factor. A random-sampling method was applied, and 107 subjects (41 male and 66 female) were selected as a control group. During the study, fortunately all the selected subjects had good cooperation with the research team, and the refusal rate was zero. Physical examination was carried out in the premise of the village's alderman in Ghopuz and in the governmental health facility in Mayan. Before physical examination, verbal consent of the participants was obtained. Table 1 presents the characteristics of the study subjects.

**Table 1.** Characteristics of study subjects: comparison between Ghopuz and Mayan

Factors assessed	Ghopuz		Mayan	
	Mean (SD)	95% CI	Mean (SD)	95% CI
CAI (g)	14.7 (10.9)	12.1–17.3	0 (0)	0
Age (years)	33.8 (16.1)	29.9–37.6	29.6 (17.9)	25.9–33.1
Residence (year)	29.7 (15.3)	26.1–33.4	28.9 (17.9)	25.3–32.5
BMI (kg/m^2^)	23.6 (4.5)	22.5–24.7	22.5 (5.8)	21.3–23.7
Systolic blood pressure (mmHg)	137 (20.2)	132–142	107 (34.9)	99.9–114
Diastolic blood pressure (mmHg)	82 (11.5)	79–85	71 (22.1)	66–75

BMI=Body mass index;

CAI=Cumulative arsenic index;

CI=Confidence interval;

SD=Standard deviation

### Sampling and analysis of water

There were three springs and a polyethylene reservoir as drinking-water sources in Ghopuz village. Water of one spring was used via the water-distribution network. However, in Mayan, well-water was available via the distribution network as the only water source. Water samples were collected in plastic bottles cleaned with nitric acid and distilled water. Two following sets of samples were collected:

The first sample was used in measuring the common parameters, including cations (Ca2+, Mg2+, Na+, K+), anions (HCO_3_-, SO_4_2-, Cl-), total dissolved solids (TDS), electrical conductivity (EC), hardness, alkalinity, and pH according to the Standard Methods for the Examination of Water and Wastewater (20th edition, 1998) (59).

The second sample was used in assessing heavy metal concentration (As, Se, Cd, Cr, Pb, Fe, Mn) in drinking-water using inductively-coupled plasma (ICP) method after acidification with nitric acid. Concentration of detection limits for each of the above elements was 70, 75, 6, 10, 64, 10, and 2 μg/L respectively. Quality control of analysis was carried out using standard solutions and plotting standard calibration curve before analysis of water samples.

To achieve a mean annual concentration of arsenic in drinking-water sources, we focused on measurement of arsenic in the sampling programme. So, measurement of arsenic was continued for one year. The results of quality analysis of drinking-water sources in Ghopuz during the past years were also collected. A structured questionnaire was used for collecting information on the type of water source, history of consumption, and changes over time.

### Definition and measurement of outcomes

Three sets of outcomes were measured, including prevalence of dermatological lesions (hyperkeratosis and pigmentation abnormalities), hypertension, and chromosomal abnormalities.

Exposure to arsenic was defined as consumption of drinking-water with arsenic concentration more than 50 μg/L for at least one year. Individuals with residency less than one year in the village were excluded from the study.

Unexposed individuals were defined as those subjects who consumed arsenic through drinking-water with a concentration of 50 μg/L or less.

After obtaining an informed consent from the participants, a specialist physician performed physical and clinical examinations. During the study, the physician was aware of exposure status. To provide a more accurate diagnosis for the skin lesions, the physicians on the research team referred to the guidelines published by the United Nations (60) and pictures available on web sources before the start of the study. During the physical examinations and after measurement of height and weight, a physician interviewed each participant for the presence of skin-lesions, discomfort, and other diseases, if any, followed by auscultation and observation of the degree of skin-hyperpigmentation, hyperkeratosis, and the severity of each lesion covering the body-surface. A questionnaire was filled for each individual during face-to-face interviews. The questionnaire contained information on age, height, weight, blood pressure, skin-lesions and its severity, location and history of these lesions, smoking, residence, and family history. We did not gather educational information and housing condition of the participants. Blood pressure of each participant was determined once in sitting position after at least 10 minutes of resting using a sphygmo portable-grade Richter manometer. Body mass index (BMI) was determined as an indicator of malnutrition status.

Cumulative arsenic index (CAI) was calculated for all the subjects (9). Lifetime dose of arsenic for each individual was then estimated on the basis of age, arsenic concentration of water sources, and location of residence using the following formula:

CAI=∑di × AIAi

AIAi=Ci × 1 g/1,000 mg × LPCD× 365 days/year

Where:

CAI is the total lifetime intake of arsenic from drinking-water (g),

di is the duration of the i^th^ water source used by each individual (in year),

AIAi is the annual intake of arsenic through consumed water in the form of water or tea (g/year),

Ci is the mean annual concentration of arsenic in drinking-water (mg/L)

LPCD (litre per capita per day) is the volume of drinking-water consumed by each individual in the form of water or tea (L/day).

### Chromosomal analysis

We investigated the occurrence of chromosomal abnormalities in subjects with chronic exposure to arsenic in drinking-water (from Ghopuz) and a matched control group with no exposure to arsenic (from Mayan). For chromosomal analysis, peripheral blood samples were assessed by micro-culture method (Difco Laboratory method), Gimsa staining, and G-banding of chromosomes. Chromosomal analysis was blinded.

### Statistical analysis

Descriptive statistics (including proportions, means, and standard deviations), 95% confidence intervals, chi-square test, and odds ratios were used for statistical analysis of data. The p value of ≤0.05 was considered significant. The SPSS software (version 10.0) was used for statistical analysis.

## RESULTS

Table 2 presents the results of analysis of water in the exposure group and control group. In the exposure group, the hardness of water was almost in the range of ‘hard to very hard’, and pH of water was more than 7. A remarkable finding in the exposure group was high amounts of nitrate in drinking-water samples taken from no-piped springs in the village (about three times more than the standard level). The results of analysis of samples from Mayan also showed the presence of minerals in water, indicating the hardness of water in the control group.

**Table 2.** Characteristics of drinking-water in Ghopuz and Mayan

Characteristics of drinking-water	Ghopuz (Exposure group)	Mayan (Control group)
Electrical conductivity (μs/cm)	1,044.8	701
pH	8.24	8.3
Total dissolved solid (mg/L)	689.8	463
Phenol alkalinity (mg/L as CaCO_3_)	2.5	0
Methyl alkalinity (mg/L as CaCO_3_)	300	185
Total hardness (mg/L as CaCO_3_)	362.5	210
Calcium (mg/L)	92.5	44
Magnesium (mg/L)	31.5	24
Sodium (mg/L)	88.9	75
Potassium (mg/L)	6.5	3.6
Chloride (mg/L)	54.9	78
Sulphate (mg/L)	83.9	70.6
Nitrite (mg/L)	0.033	0
Nitrate (mg/L)	111.6	8.5
Iron (mg/L)	0.0465	0.102
Manganese (mg/L)	0.001	0.002
Cadmium (mg/L)	0	0
Arsenic (mg/L)	1.031*	0
Selenium (mg/L)	0.0075	0.007
Chromium (mg/L)	0	0
Lead (mg/L)	0	0

*Mean of 4 drinking-water sources during one year of measurements

Arsenic contamination was detected in water sources of the exposed subjects. Analyses of other elements showed a slightly higher concentration of selenium in the exposure group. In the water samples from Mayan (control group), we did not find any amount of arsenic. Interestingly, there was no high amount of manganese, along with arsenic.

General characteristics of the study subjects are presented in Table 1. No significant effect was observed for cigarette-smoking (p=0.55). The mean systolic blood pressure in Ghopuz (n=137, 95% CI 132–142) was significantly higher than from the control subjects (n=107, 95% CI 99.9–114). The same difference was observed for diastolic blood pressure (Ghopuz: n=82, 95% CI 79–85; Mayan: n=71, 95% CI 66–75).

Table 3 shows a comparison of dermatological lesions and chromosomal abnormalities in the exposure group and control group. The incidence of hyperkeratosis was 34 times higher among the exposed individuals compared to those in the control group (OR=34, p<0.001). The same difference was found in the location and severity of the lesions. Palmar hyperkeratosis was observed in one subject in each group (p>0.1).

**Table 3.** Dermatological lesions and chromosomal abnormalities in Ghopuz and Mayan

Disorder	Ghopuz		Mayan		Statistical significance
	No.	%	No.	%	
Keratosis					
No	68	68.7	105	99.1	OR=34, p<0.001
Yes	31	31.3	1	0.9	
Location of keratosis					
No keratosis	68	68.7	105	99.1	
Hands	1	1	1	0.9	OR=1.1, p>0.10
Feet	21	21.2	0	0	p<0.001
Both hands and feet	9	9.1	0	0	p<0.001
Severity of keratosis					
No keratosis	68	68.7	105	99.1	
Mild	21	21.2	1	0.9	OR=23, p<0.001
Moderate	10	10.1	0	0	p<0.001
Pigmentation					
No	77	77	97	90.7	
Yes	23	23	10	9.3	OR=2.4, p=0.007
Location of pigmentation					
No pigmentation	77	77	97	90.7	
Limbs	0	0	1	0.9	p<0.001
Limbs and trunk	21	21	0	0	p<0.001
Limbs, trunk, and tongue	2	2	9	8.4	OR=0.24, p=0.17
Severity of pigmentation					
No pigmentation	77	77	97	92.4	
Mild	8	8	4	3.8	OR=2.1, p<0.001
Moderate	14	14	4	3.8	OR=3.7, p<0.001
Severe	1	1	0	0	p<0.001
Chromosomal abnormalities					
No abnormalities	18	75	18	81.8	
21 Trisomy	0	0	2	9.1	
Chromatide cleavage	0	0	2	9.1	
Endo-reduplication	2	8.3	0	0	
Endo-reduplication, gap and acentric fragment	1	4.1	0	0	p<0.05
Acentric fragment and 45- (-x or -C)/47- chromosomes	3	12.5	0	0	

OR=Odds ratio

A significant difference was found in the occurrence of skin-pigmentation, location and severity of pigmentations between the two groups (OR=2.4, p<0.007). Concurrent pigmentation in limbs, trunk, and tongue was observed in two of the exposed subjects and nine in the controls. This difference was not statistically significant (OR=0.24, p=0.17).

## DISCUSSION

Cutaneous abnormalities and lesions are the most common outcome and health effects from consumption of drinking-water containing arsenic. Typically, the cutaneous abnormalities and lesions are diagnosed as keratosis and pigment disorder, including hyperpigmentation and hypopigmentation. We studied the effects of chronic exposure to arsenic on dermatological lesions, hypertension and chromosomal abnormalities in a region in the northwest of Iran. The findings showed a high prevalence of skin disorders in individuals who consumed water with high concentrations of arsenic.

The results of the study showed that, in the arsenic-affected village, 30 (30.6%) subjects had hyperkeratosis, and 23 (23%) subjects had hyperpigmentation. However, in the control village, we found one person (0.9%) with keratosis and 10 persons (9.3%) with pigmentation.

A study at Guo in Inner Mongolia found that the prevalence of keratosis was higher than pigment disorder. There was a clear exposure-response relationship between the prevalence of pigmented lesions and the levels of arsenic (61).

Mosaferi *et al*. studied 752 subjects in Kurdistan province of Iran in eight villages of Bijar county. They found a significant correlation of chronic arsenic exposure with hyperkeratosis and hyperpigmentation. There was also a significant relationship between total lifetime intake of arsenic and keratosis, pigmentation, systolic and diastolic blood pressures, and hair arsenic content (9, 62).

In the study of Tondel in Bangladesh, morbidity from skin-lesions included almost one-third of the population which is similar to our study (63).

Cumulative arsenic index (CAI) has been shown to be associated with hypertension in a dose-response. This index is able to well-reflect the cumulative dosage of lifetime exposure to arsenic via drinking-water in individual subjects.

The study of Huang further explored the association between arsenic methylation capability and hypertension risk among residents of arseniasis hyper-endemic areas in Taiwan (64). The findings suggested that hypertensive subjects had higher urinary monomethylarsonic acid (MMA^V^) percentage and lower secondary arsenic methylation index (SMI) than subjects without hypertension (64).

Other investigations in Taiwan and Bangladesh have shown that subjects with prolonged exposure to inorganic arsenic have a significantly higher risk of hypertension in a dose-response pattern (27, 28).

A cross-sectional study on 8,790 pregnant women observed increased systolic blood pressure levels with increasing arsenic contents in drinking-water (65). Our study has shown that subjects with high CAI had higher blood pressure even after stopping consumption of contaminated water for five years.

In Iran, the delayed effects of exposure to arsenic on health, incomplete monitoring of chemical quality, technical limitation for analysis of arsenic, the lack of common definitions, poor awareness, and reporting in the region are the major problems in determining the extent of the arsenic problem in drinking-water. It seems, therefore, necessary to create a reliable databank and information system for chemical quality of drinking-water in some suspicious polluted areas in the country.

Our study had some limitations. The physician was aware of exposure status. Determination of skin-manifestation in the exposure group and control group was not, therefore, blind. Blood pressure was measured once only while it is recommended to repeat the measurement 2–3 times for a valid result. For calculation of CAI value, it was difficult to obtain and calculate accurate data of water consumption for each individual and the duration of exposure to arsenic.

In Ghopuz village, an important concern was the consumption of contaminated water by livestock because of the shortage of safe water in the region. Results of a study in Argentina showed that contamination in water supply for livestock may lead to contamination of dairy (66). This emphasizes the necessity of monitoring the chemical profile of water sources in livestock too, even if there is no detectable/considerable contamination in water supply for humans. Another important issue in dealing with arsenic-related problems is public education, particularly in the communities with low socioeconomic status.

The study concludes that arsenic exposure is a serious health problem in the region. More studies are needed to investigate the long-term effects and dose-response relationship of arsenic in the region and similar areas. Wide-ranging monitoring programmes of drinking-water sources should be implemented by public-health authorities.

Geo-coding of contaminated areas (using the Geographical Information System) and monitoring the contamination rate of soil and agricultural products in arsenic-contaminated areas may also help prevent further similar problems in the region.

## ACKNOWLEDGEMENTS

The authors thank the East Azerbaijan Sciences and Technology Park for funding this project. The authors also thank all the participants in this project in East Azerbaijan for their hospitality and assistance during the study and data collection. They are grateful of Dr. Kusha and Dr. Seyf Farshad for their help in coordination and management of the project.

## References

[1] Agency for Toxic Substances and Disease Registry (2005). Toxicological profile for arsenic TP2.

[2] World Health Organization (2001). Arsenic and arsenic compounds, 2nd ed..

[3] Rousselot P, Laboume S, Marolleau JP, Larghero T, Noguera ML, Brouet JC (1999). Arsenic trioxide and melarsoprol induce apoptosis in plasma cell lines and in plasma cells from myeloma patient. Cancer Res.

[4] Evens AM, Tallman MS, Gartenhaus RB (2004). The potential of arsenic trioxide in the treatment of malignant disease: past, present, and future. Leukemia Res.

[5] Petrusevski B, Sharma SK, Schippers JC, Shordt K (2007). Arsenic in drinking water.

[6] Tseng CH (2008). Cardiovascular disease in arsenic-exposed subjects living in the arseniasis-hyperendemic areas in Taiwan (review). Atherosclerosis.

[7] Rahman MM, Chowdhury UK, Mukherjee SC, Mondal BK, Paul K, Lodh D (2001). Chronic arsenic toxicity in Bangladesh and West Bengal, India, a review and commentary. J Toxicol Clin Toxicol.

[8] Mukherriee A, Sengupta MK, Hossain MA, Ahamed S, Das B, Navak B (2006). Arsenic contamination in groundwater: a global perspective with emphasis on the Asian scenario. J Health Popul Nutr.

[9] Mosaferi M, Yunesian M, Dastgiri S, Mesdaghinia A, Esmailnasab N (2008). Prevalence of skin lesions and exposure to arsenic in drinking water in Iran. Sci Total Environ.

[10] Mosaferi M, Hassani AM, Borghei M, Kamali Z, Ghadirzadeh A (2008). Study of arsenic presence in drinking water sources: a case study. Iran J Health Environ.

[11] World Health Organization (1993). Guidelines for drinking-water quality.

[12] Institute of Standard and Industrial Research of Iran (1996). Drinking water characteristics. Standard number 1053.

[13] Uchino T, Roychowdhury T, Ando M, Tokunaga H (2006). Intake of arsenic from water, food composites and excretion through urine, hair from a studied population in West Bengal, India. Food Chem Toxicol.

[14] Yoshida T, Yamauchi H, Sun GF (2004). Chronic health effects in people exposed to arsenic via the drinking water-dose response relationships in review. Toxicol Appl Pharmacol.

[15] Nickson R, McArthur J, Burgess W, Ahmed KM, Ravenscroft P, Rahman M (1998). Arsenic poisoning of Bangladesh groundwater (letter). Nature.

[16] Gebel TW (1999). Arsenic and drinking water contamination. Science.

[17] Sun G (2004). Arsenic contamination and arsenicosis in China. Toxicol Appl Pharmacol.

[18] Kapaj S, Peterson H, Liber K, Bhattacharya P (2006). Human health effects from chronic arsenic poisoning: a review. J Environ Sci Health A: Tox Hazard Subst Environ Eng.

[19] Tsuchiya K (1977). Various factors influencing toxicity and metabolism of metals–metal-metal interactions and host factors [author's transl]. Sangyo Igaku.

[20] Ferreccio C, Sancha AM (2006). Arsenic exposure and its impact on health in Chile. J Health Popul Nutr.

[21] Mitra SR, Mazumder DN, Basu A, Block G, Haque R, Samanta S (2004). Nutritional factors and susceptibility to arsenic-caused skin lesions in West Bengal, India. Environ Health Perspect.

[22] Kristiansen J, Christensen JM, Iversen BS, Sabbioni E (1997). Toxic trace element reference levels in blood and urine: influence of gender and lifestyle factors. Sci Total Environ.

[23] Navas-Acien A, Sharret AR, Silbergeld EK, Schwartz BS, Nachman KE, Burke TA (2005). Arsenic exposure and cardiovascular disease: a systematic review of the epidemiologic evidence. Am J Epidemiol.

[24] Tseng CH, Chong CK, Tseng CP, Hsueh YM, Chiou HY, Tseng CC (2003). Long-term arsenic exposure and ischemic heart disease in arseniasis-hyperendemic villages in Taiwan. Toxicol Lett.

[25] Chiou HY, Huang WI, Su CL, Chang SF, Hsu YH, Chen CJ (1997). Dose-response relationship between prevalence of cerebrovascular disease and ingested inorganic arsenic. Stroke.

[26] Wang CH, Jeng JS, Yip PK, Chen CL, Hsu LI, Hsueh YM (2002). Biological gradient between long-term arsenic exposure and carotid atherosclerosis. Circulation.

[27] Chen CJ, Hsueh YM, Lai MS, Shyu MP, Chen SY, Wu MM (1995). Increased prevalence of hypertension and long-term arsenic exposure. Hypertension.

[28] Rahman M, Tondel M, Ahmad SA, Chowdhury IA, Faruquee MH, Axelson O (1999). Hypertension and arsenic exposure in Bangladesh. Hypertension.

[29] Engel RR, Hopenhayn-Rich C, Receveur O, Smith AH (1994). Vascular effects of chronic arsenic exposure: a review. Epidemiol Rev.

[30] Simeonova PP, Luster MI (2004). Arsenic and atherosclerosis. Toxicol Appl Pharmacol.

[31] Tseng CH, Chong CK, Chen CJ, Tai TY (1996). Dose-response relationship between peripheral vascular disease and ingested inorganic arsenic among residents in blackfoot disease endemic villages in Taiwan. Atherosclerosis.

[32] Kumagai Y, Pi J (2004). Molecular basis for arsenic-induced alteration in nitric oxide production and oxidative stress: implication of endothelial dysfunction. Toxicol Appl Pharmacol.

[33] Tseng WP (1989). Blackfoot disease in Taiwan: a 30-year follow-up study. Angiology.

[34] Chen CJ, Wang CJ (1990). Ecological correlation between arsenic level in well water and age-adjusted mortality from malignant neoplasms. Cancer Res.

[35] Chen CJ, Chuang YC, Lin TM, Wu HY (1985). Malignant neoplasms among residents of a blackfoot disease-endemic area in Taiwan: high-arsenic artesian well water and cancers. Cancer Res.

[36] Chen CJ, Chen CW, Wu MM, Kuo TL (1992). Cancer potential in liver, lung, bladder and kidney due to ingested inorganic arsenic in drinking water. Br J Cancer.

[37] Chiou HY, Chiou ST, Hsu YH, Chou YL, Tseng CH, Wei ML (2001). Incidence of transitional cell carcinoma and arsenic in drinking water: a follow-up study of 8102 residents in an arseniasis-endemic area in northeastern Taiwan. Am J Epidemiol.

[38] Schwerdtle T, Walter I, Mackiw I, Hartwig A (2003). Induction of oxidative DNA damage by arsenite and its trivalent and pentavalent methylated metabolites in cultured human cells and isolated DNA. Carcinogenesis.

[39] Yu HS, Liao WT, Chai CY (2006). Arsenic carcinogenesis in the skin. J Biomed Sci.

[40] Chiou HY, Hsueh YM, Liaw KF, Horng SF, Chiang MH, Pu YS (1995). Incidence of internal cancers and ingested inorganic arsenic: a seven-year follow-up study in Taiwan. Cancer Res.

[41] Liu YT, Chen Z (1996). A retrospective lung cancer mortality study of people exposed to insoluble arsenic and radon. Lung Cancer.

[42] Abernathy C, Morgan A (2001). Exposure and health effects. Chapter 3: United Nations synthesis report on arsenic in drinking water (first draft).

[43] Tapio S, Grosche B (2006). Arsenic in the aetiology of cancer. Mutation Res.

[44] Navas-Acien A, Silbergeld EK, Streeter RA, Clark JM, Burke TA, Guallar E (2006). Arsenic exposure and type 2 diabetes: a systematic review of the experimental and epidemiologic evidence. Environ Health Perspect.

[45] Tseng CH, Tseng CP, Chiou HY, Hsueh YM, Chong CK, Chen CJ (2002). Epidemiologic evidence of diabetogenic effect of arsenic. Toxicol Lett.

[46] Calderon J, Navarro ME, Jimenez-Capdeville ME, Santos-Diaz MA, Golden A, Rodriguez-Leyva I (2001). Exposure to arsenic and lead and neuropsychological development in Mexican children. Environ Res.

[47] Grandjean P, Landrigan PJ (2006). Developmental neurotoxicity of industrial chemicals. Lancet.

[48] Yoshida T, Yamauchi H, Fan Sun G (2004). Chronic health effects in people exposed to arsenic via the drinking water: dose-response relationships in review. Toxicol Appl Pharmacol.

[49] Mazumder DNG (2003). Chronic arsenic toxicity: clinical features, epidemiology, and treatment: experience in West Bengal. J Environ Sci Health A: Tox Hazard Subst Environ Eng.

[50] Tseng WP (1977). Effects and dose–response relationships of skin cancer and blackfoot disease with arsenic. Environ Health Perspect.

[51] Wang YH, Wu MM, Hong CT, Lien LM, Hsieh YC, Tseng HP (2007). Effects of arsenic exposure and genetic polymorphisms of p53, glutathione S-transferase M1, T1, and P1 on the risk of carotid atherosclerosis in Taiwan. Atherosclerosis.

[52] Mahata J, Chaki M, Ghosh P, Das LK, Baidya K, Ray K (2004). Chromosomal aberrations in arsenic-exposed human populations: a review with special reference to a comprehensive study in West Bengal. India. Cytogenet Genome Res.

[53] Sordo M, Herrera LA, Ostrosky-Wegman P, Rojas E (2001). Cytotoxic and genotoxic effects of As, MMA, and DMA on leukocytes and stimulated human lymphocytes. Teratog Carcinog Mutagen.

[54] Abernathy CO, Liu YP, Longfellow D, Aposhian HV, Beck B, Fowler B (1999). Arsenic: health effects, mechanisms of actions, and research issues. Environ Health Perspect.

[55] Dopp E, Hartmann LM, Florea AM, von Recklinghausen U, Pieper R, Shokouhi B (2004). Uptake of inorganic and organic derivatives of arsenic associated with induced cytotoxic and genotoxic effects in Chinese hamster ovary (CHO) cells. Toxicol Appl Pharmacol.

[56] Hughes MF (2002). Arsenic toxicity and potential mechanisms of action. Toxicol Lett.

[57] Chen CJ, Hsueh YM, Chiou HY, Abernathy CO, Calderon RL, Chappell WR (1997). Human carcinogenicity of inorganic arsenic. Arsenic: exposure and health effects.

[58] Iran national portal of statistics (2008). http://www.sci.org.ir/portal/faces/public/sci.

[59] Clesceri LS, Greenberg AE, Eaton AD (1998). Standard methods for the examination of Water and wastewater.

[60] Guha Mazumder DN (2000). Diagnosis and treatment of chronic arsenic poisoning.

[61] Guo X, Liu Z, Huang C, You L (2006). Levels of arsenic in drinking water and cutaneous lesions in Inner Mongolia. J Health Popul Nutr.

[62] Mosaferi M, Yunesian M, Mesdaghinia AR, Nasseri S, Mahvi AH, Nadim H (2005). Correlation between arsenic concentration of drinking water and hair. Iran J Environ Health Sci Eng.

[63] Tondel M, Rahman M, Magnuson A, Chowdhury IA, Faruquee MH, Ahmad SA (1999). The relationship of arsenic levels in drinking water and the prevalence rate of skin lesions in Bangladesh. Environ Health Perspect.

[64] Huang YK, Tseng CH, Huang YL, Yang MH, Chen CJ, Hsueh YM (2007). Arsenic methylation capability and hypertension risk in subjects living in arseniasis-hyperendemic areas in southwestern Taiwan. Toxicol Appl Pharmacol.

[65] Ling HL, Xia Y, Lobdell D, Zeng D, Thorp JM, Creason JP (2007). Drinking water arsenic exposure and blood pressure in healthy women of reproductive age in Inner Mongolia, China. Toxicol Appl Pharmacol.

[66] Perez-Carrera A, Fernandez-Cirelli A (2005). Arsenic concentration in water and bovine milk in Cordoba, Argentina: preliminary results. J Dairy Res.

